# Myoglobin and left ventricular ejection fraction as predictive markers for death in children with fulminant myocarditis

**DOI:** 10.3389/fped.2022.949628

**Published:** 2022-09-14

**Authors:** Tingting Xie, Xiaodong Zang, Yingying Xiong, Chaolei Yang, Fei Li, Dandan Wang, Yaqin Shu, Xuming Mo, Mingwu Chen

**Affiliations:** ^1^Department of Pediatrics, Provincial Hospital Affiliated to Anhui Medical University, Hefei, China; ^2^Division of Life Sciences and Medicine, Department of Pediatrics, The First Affiliated Hospital of Science and Technology of China, University of Science and Technology of China (USTC), Hefei, China; ^3^Department of Cardiothoracic Surgery, Children's Hospital of Nanjing Medical University, Nanjing, China

**Keywords:** fulminant myocarditis, myoglobin, left ventricular ejection fraction, children, prognosis

## Abstract

**Background:**

Fulminant myocarditis (FM) is an inflammatory process of the myocardium and an important cause of cardiac dysfunction in children; it is characterized by rapid onset, acute progression, and high mortality. The study sought to describe the clinical characteristics and prognostic factors in children with FM.

**Methods:**

The study population consists of 37 consecutive patients admitted from May 2014 to December 2021 with a diagnosis of FM. According to the prognosis of children with FM during hospitalization, they were divided into “survival” group (25 cases) and “death” group (12 cases). A multivariate logistic regression analysis was performed to identify the independent predictors of in-hospital mortality in the patients, and receiver operating characteristic (ROC) curve was used to explore the predictive value of related factors.

**Results:**

The 37 children with FM had an average age of 8.35 ± 4.36 years old. Twenty-five of the patients survived and 12 died. Twenty-five of the children were discharged from the hospital after a series of active rescue treatments such as nutritional myocardial drugs, high-dose intravenous immunoglobulin (IVIG), glucocorticoids (GCs), temporary pacemaker (TP), extracorporeal membrane oxygenation (ECMO), and continuous renal replacement therapy (CRRT).Twelve of the children were classified into the death group because the resuscitation failed. The levels of procalcitonin (PCT), creatine kinase (CK), and myoglobin (MYO) in the death group were all higher than in the survival group (all *P* < 0.05), and the left ventricular ejection fraction (LVEF) in the death group was significantly lower than in the survival group (*P* = 0.002). The binary logistic regression analysis revealed that MYO [OR:1.006; 95%CI:(1–1.012); *P* = 0.045] and LVEF [OR: 0.876; 95% CI: (0.785–0.978); *P* = 0.019] were independent predictors of FM. ROC curve analysis showed that the area under ROC curve (AUC) of MYO and LVEF was [AUC:0.957; 95%CI:0.897~1] and [AUC:0.836; 95%CI:0.668~1], and the area under the combined ROC curve for MYO + LVEF was significantly higher than that for MYO or LVEF alone (*P* < 0.05), indicating that the MYO + LVEF combined diagnosis had a higher predictive value for FM.

**Conclusion:**

The levels of MYO and LVEF can be markers for prognosis of FM and can effectively evaluate the disease severity. Their combination can improve forecast accuracy; thus, the detection of the above-mentioned indexes possesses a higher value for clinical applications.

## Introduction

As one of the most serious types of acute myocarditis in children, FM is a rapidly progressing clinical syndrome whose dramatic presenting scenarios include rapidly progressive hemodynamic compromise and fatal arrhythmia, resulting in cardiogenic shock or even sudden death (1, 2). The early clinical symptoms of FM are nonspecific, such as fever, fatigue, and chest tightness, making diagnosis difficult and leading to high incidence of misdiagnosis, which leads to high clinical mortality (3). Recently, a retrospective study published by the American College of Cardiology showed that FM has a high in-hospital mortality rate of 25.5% (4). Hence, it is particularly important to find the predictors of death in FM and intervene early.

The previous study suggested an important role of histological classification of FM, and the histological subtype of FM was a determinant of outcomes (5). Besides, LVEF, glomerular filtration rate (GFR), left atrial diameter (LAD), and ACEF score, may provide a predictive value for the prognosis of FM (6, 7). It was previously demonstrated that MYO may have better prognostic performance than other cardiac markers in COVID-19 (8). According to latest research, patients with new-onset acute heart failure with reduced ejection fraction had a good prognosis (9). However, its prognostic value in patients with FM is still unknown. Thus, our study aimed to investigate the prognostic value of MYO and LVEF in patients with FM.

## Materials and methods

### Study population

**Figure 1:**
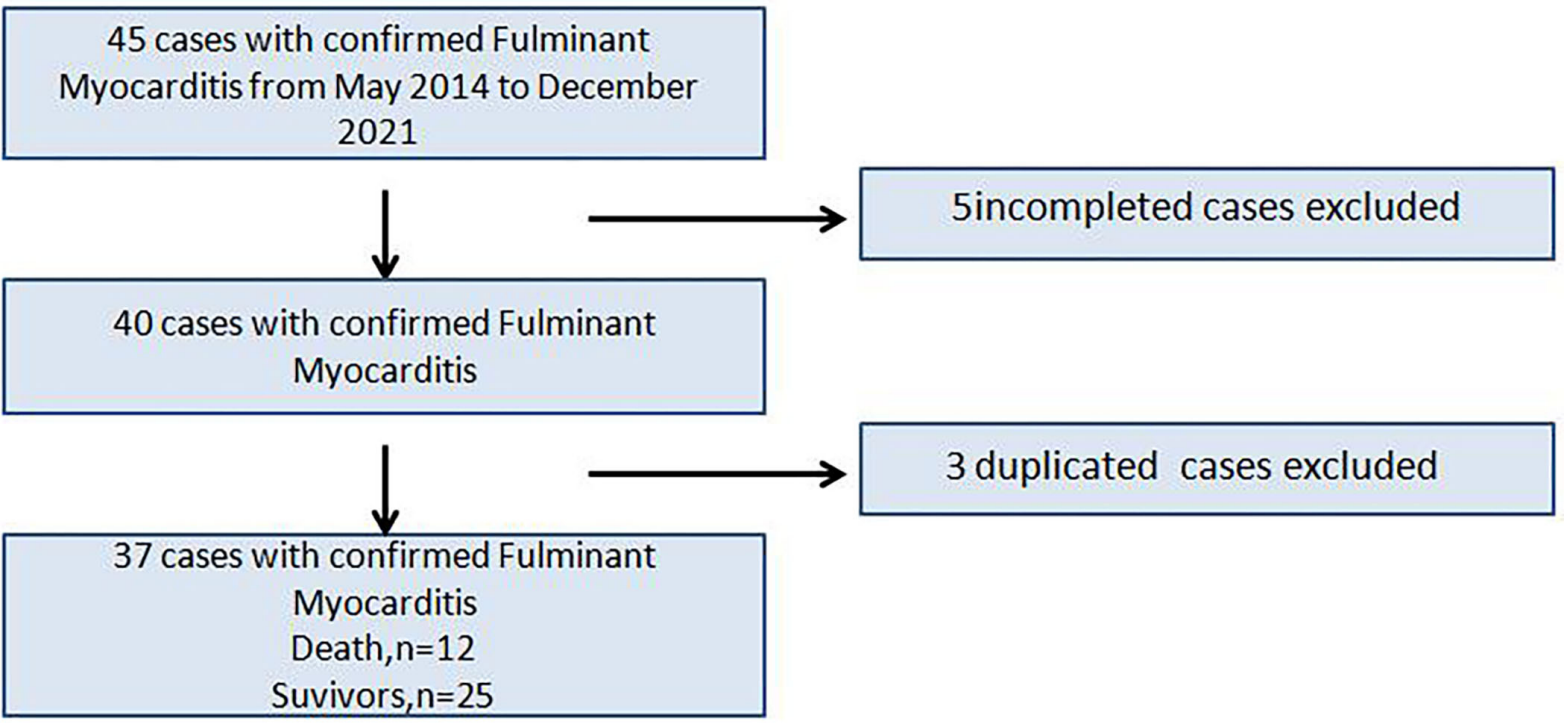
Flowchart of patient recruitment.

Forty five children were admitted to the First Affiliated Hospital of USTC, Division of Life Science and Medicine, University of Science and Technology of China (Anhui Provincial Hospital) and Children's Hospital of Nanjing Medical University. 5 cases with incomplete data and 3 cases with repeated medical records were excluded, and finally 37 children were retrospectively analyzed (Figure 1). This study was retrospective, and had been approved by the hospital ethics committee to exempt patients' informed consent.

### Inclusion criteria

All the children with FM met the diagnostic criteria for myocarditis in the Diagnostic Recommendation For Myocarditis In Children (version 2018) (10). The patient inclusion criteria were as follows: (A) sudden onset, (B) hemodynamic instability due to cardiogenic shock or arrhythmia, (C) hemodynamic or circulatory support to maintain heart function, and (D) evidence of myocardial damage suggesting cardiac dysfunction such as changes in CK-MB levels, CTnI levels, ECG, and echo.

### Exclusion criteria

Children with congenital heart disease, endocardial elastic fibrosis, myocardial infarction, non-ischemic cardiomyopathy, and genetic disease were excluded from this study.

### Data collection

The demographic data, vital signs, clinical presentation, laboratory indexes, ECG manifestations, and echo results and the treatment regimen of each child were collected and reviewed. The demographics included age, sex, family history, and past history. The basic vital signs included temperature, systolic blood pressure (SBP), diastolic blood pressure (DBP), respiration, and pulse on admission.

Clinical manifestations are mainly a series of uncomfortable reactions of a patient, including chest tightness, chest pain, dyspnea, fever, and other common features of FM, as well as other clinical manifestations of the patient's respiratory system, digestive system, and nervous system.

The laboratory indexes included routine tests such as blood routine, biochemistry, liver and kidney functions, especially indicators reflecting cardiac function including myoglobin (MYO), creatine kinase MB (CK-MB), high-sensitivity troponin I (CTnI), B-type natriuretic peptide (BNP), and creatine kinase (CK),which were determined with an OLYMPUS automatic biochemical analyzer (AU2700: Japan).

ECG parameters included the type of arrhythmiathe, change of ST segment and the prolongation of QT interval, which were collected by Foton FX-7404 electrocardiograph with standard 12-lead electrocardiogram.

The echo mainly recorded LVEF, valvular regurgitation, pericardial effusion, cardiac enlargement, and systolic and diastolic dysfunctions, which were detected with PHILIPS EPIQ 7C color Doppler.

### Statistical analysis

Continuous variables with a normal distribution are expressed as the means ± standard deviation (x ± s), and between-group comparisons are performed by independent samples *t*-test. Continuous variables for partial distributions are expressed as medians (quartile interval) [M (Q1, Q3)], and between-group comparisons are performed by non-parametric test. Count data are expressed as the number of cases (%), and the difference between groups is compared by chi-squared test or Fisher's exact test. A two-tailed *P* < 0.05 is set as statistically significant. A multivariate logistic regression analysis is performed to identify the independent predictors of in-hospital mortality in the patients, and receiver operating characteristic (ROC) curve is used to explore the predictive value of related factors. Spearman correlation analysis tests the association between MYO and inflammatory biomarkers. A statistical analysis is performed using SPSS 28.0.

## Results

### Clinical characteristics

Among the 37 children with FM, 16 were males (43.2%) and 21 were females (56.8%). The age distribution was as follows: 5 patients were from 0 to 3 years old, 11 were from 4 to 7 years old, 15 were from 8 to 12 years old, and 6 were from 13 to 17 years old.

The patients were divided into two groups: those that survived to discharge (“survival” group, 25 cases) and those that died while in the hospital (“death” group, 12 cases), and risk factors for in-hospital death were analyzed.

The demographic data, vital signs, clinical manifestations, laboratory data, ECG manifestations, and echo measurements on admission are shown in Tables 1–3.

**Table 1:** Comparison of basic conditions and clinical manifestations of children with FM.

**Characteristic**	**Survivors** **(*n* = 25)**	**Death** **(*n* = 12)**	***P***
Demographics			
Gender [man, *n* (%)]	11 (44)	5 (41.7)	0.893
Age [years, M (QI, Q3)]	8 (5,12)	10 (4,11)	0.896
Hospital time [day, M (QI, Q3)]	31 (18,48)	9 (2,20)	0.002
Vital Signs			
SBP[mmHg, M (QI, Q3)]	92 (88,102)	97 (85,113)	0.812
DBP [mmHg, M (QI, Q3)]	60 (55,65)	54 (47,60)	0.207
Pulse [F/min, M (QI, Q3)]	110 (70,125)	129 (83,170)	0.168
Breathe [F/min, M (QI, Q3)]	26 (20,36)	27 (20,36)	0.960
Symptoms			
Chest tightness [*n* (%)]	13 (52)	6 (50)	0.909
Chest pain [*n* (%)]	4 (16)	1 (8.3)	0.901
Difficulty breathing [*n* (%)]	6 (24)	2 (16.7)	0.936
Palpitations [*n* (%)]	2 (8)	1 (8.3)	1.000
Fatigue [*n* (%)]	2 (8)	0	0.817
Fever [*n* (%)]	14 (56)	7 (58.3)	0.893
Respiratory system [*n* (%)]	10 (40)	3 (25)	0.598
Digestive system [*n* (%)]	17 (68)	5 (41.7)	0.127
Nervous system [*n* (%)]	5 (20)	6 (50)	0.062

SBP, systolic blood pressure; DBP, diastolic blood pressure.

**Table 2:** Comparison of laboratory parameters in patients with FM.

**Characteristic**	**Survivors** **(*n* = 25)**	**Death** **(*n* = 12)**	***P***
CRP [mg/L, M (QI, Q3)]	22.39 (11.30,46.50)	66.82 (9.24,110.10)	0.136
PCT [ng/ml, M (QI, Q3)]	0.188 (0.100,0.420)	0.750 (0.376,2.695)	0.006
BUN [mmol/L, M (QI, Q3)]	6.6 (5,10.8)	7 (4.7,13)	0.932
CR [μmol/L, M (QI, Q3)]	53 (39.8,74)	59 (35.6,112.0)	0.430
ALT [IU/L, M (QI, Q3)]	42.3 (25.0,151.2)	124.5 (44.0,313.0)	0.153
AST [IU/L, M (QI, Q3)]	136 (83,359.1)	396.7 (195,611.3)	0.189
CK-MB [IU/L, M (QI, Q3)]	69 (53,105)	110.23 (61.64,198)	0.096
CK [IU/L, M (QI, Q3)]	765 (606,1083)	1412 (1042,2296)	0.038
TNIU [μg/L, M (QI, Q3)]	10.72 (3.68,22.70)	13.87 (6.25,40.36)	0.381
NTproBNP [pg/ml, M (QI, Q3)]	4186 (1093,12685)	2954 (1781,6570)	0.897
MYO [μg/L, M (QI, Q3)]	109.4 (52.2,171.3)	334.36 (237.55,1430.55)	<0.001

CRP, C reactive protein; PCT, procalcitonin; BUN, urea nitrogen; CR, creatinine; ALT, alanine transaminase; AST, aspartate transaminase; CK-MB, creatine kinase-MB; CK, creatine kinase; TNIU, troponin I; NTproBNP, N-terminal natriuretic peptide precursor; MYO, myoglobin.

**Table 3:** Comparison of ECHO and ECG of children with FM.

**Characteristic**	**Survivors** **(*n* = 25)**	**Death** **(*n* = 12)**	***P***
Echocardiography			
LVEF [%,M(QI,Q3)]	44 (36,59)	24 (20,36)	0.002
MR [*n* (%)]	18 (72)	9 (75)	0.847
TR [*n* (%)]	15 (60)	7 (58.3)	0.923
AR [*n* (%)]	10 (40)	5 (41.7)	0.923
PH [*n* (%)]	7 (28)	2 (16.7)	0.732
PE [*n* (%)]	9 (36)	1 (8.3)	0.168
CCE [*n* (%)]	14 (56)	5 (41.7)	0.414
LVSD [*n* (%)]	11 (44)	8 (66.7)	0.347
LVDD [*n* (%)]	2 (8.0)	0	1.000
RIWM [*n* (%)]	9 (36)	4 (33.3)	1.000
Electrocardiogram			
Tachycardia [*n* (%)]	16 (64)	8 (66.7)	1.000
Conduction block [*n* (%)]	8 (32)	4 (33.3)	1.000
Preterm contraction [*n* (%)]	2 (8.0)	1 (8.3)	1.000
ST-T change [*n* (%)]	11 (44)	5 (41.7)	0.893
Abnormal Q [*n* (%)]	5 (20)	2 (16.7)	1.000
Electric axis offset [*n* (%)]	7 (28)	1 (8.3)	0.350
Long QT interval [*n* (%)]	1 (4.0)	0	1.000

LVEF, left ventricular ejection fraction; MR, mitral regurgitation; TR, tricuspid regurgitation; AR, aortic regurgitation; PH, pulmonary hypertension; PE, pericardial effusion; CCE, cardiac chamber enlargement; LVSD, left ventricular systolic dysfunction; RIWM, reduction in wall motion; LVDD, left ventricular diastolic dysfunction.

The children with FM were mostly concentrated in two age groups, 4–7 and 8–12 years old, and the morbidity and mortality of children aged 8–12 years were relatively high. Gender differences in children with FM were not significant, and morbidity and mortality were higher in women.

Furthermore, we found that the main clinical symptoms of the children with FM were chest tightness, chest pain, fever, dyspnea, fatigue, and other symptoms. Compared with the survival group, the children of the death group had a higher frequency of abnormal neurological manifestations such as headache, syncope, etc.

Among the cardiac function indexes, the CK and MYO levels of children in death group were higher than those in the survival group, and the difference were statistically significant (*P* < 0.05). The rest was not statistically significant.

The ECG manifestations of the children with FM were tachycardia and conduction block, often accompanied by changes in ST-T. Compared with that in the survival group, the level of LVEF in the death group was significantly lower (*P* = 0.002).

FM mainly relied on supportive and symptomatic therapy. Initial treatment often required mechanical ventilation, inotropic agents, and vasopressors to correct hypotension, respiratory failure, and overt cardiogenic shock. For critically ill patients, a series of circulatory and renal replacement therapy was required.

The univariate analysis showed an index of *P* < 0.05, and the binary logistic regression analysis showed that MYO [OR: 1.006; 95% CI: (1~1.012); *P* = 0.045] and LVEF [OR: 0.876; 95% CI: (0.785~0.978); *P* = 0.019] were important predictors of death in children with FM (Table 4).

**Table 4:** Binary logistic regression analysis of predictors of in-hospital mortality in children with FM.

**Variables**	**Odds ratio (95%CI)**	***P* value**
MYO	1.006 (1.000~1.012)	0.045
LVEF	0.876 (0.785~0.978)	0.019

The ROC curve analysis showed that MYO [AUC: 0.957; 95% CI: 0.897 ~1], LVEF [AUC: 0.836; 95% CI: 0.668~1], and the combination of MYO and LVEF [AUC: 0.972; 95% CI: 0.916~1] significantly improved the diagnostic efficiency for FM. The sensitivity and specificity of the two combined to predict the prognosis of FM were 100 and 96% (Table 5), respectively. The combined prediction of MYO and LVEF, which can effectively improve the sensitivity and accuracy of clinical diagnosis, reduces the probability of missed diagnosis and misdiagnosis, and provides a reference for clinical treatment plan and prognosis.

**Table 5:** ROC analysis of MYO, LVEF, and combination in the diagnosis of FM in-hospital death.

**Variables**	**AUC (95% CI)**	**Cut-off level**	**Sensitivity (%)**	**Specificity (%)**	***P* value**
MYO	0.957(0.897~1.000)	210	91.7	88	0
LVEF	0.836(0.668~1.000)	31.5	70	96	0.002
MYO and LVEF	0.972(0.916~1.000)	0.2513221	100	96	0

**Figure 2:**
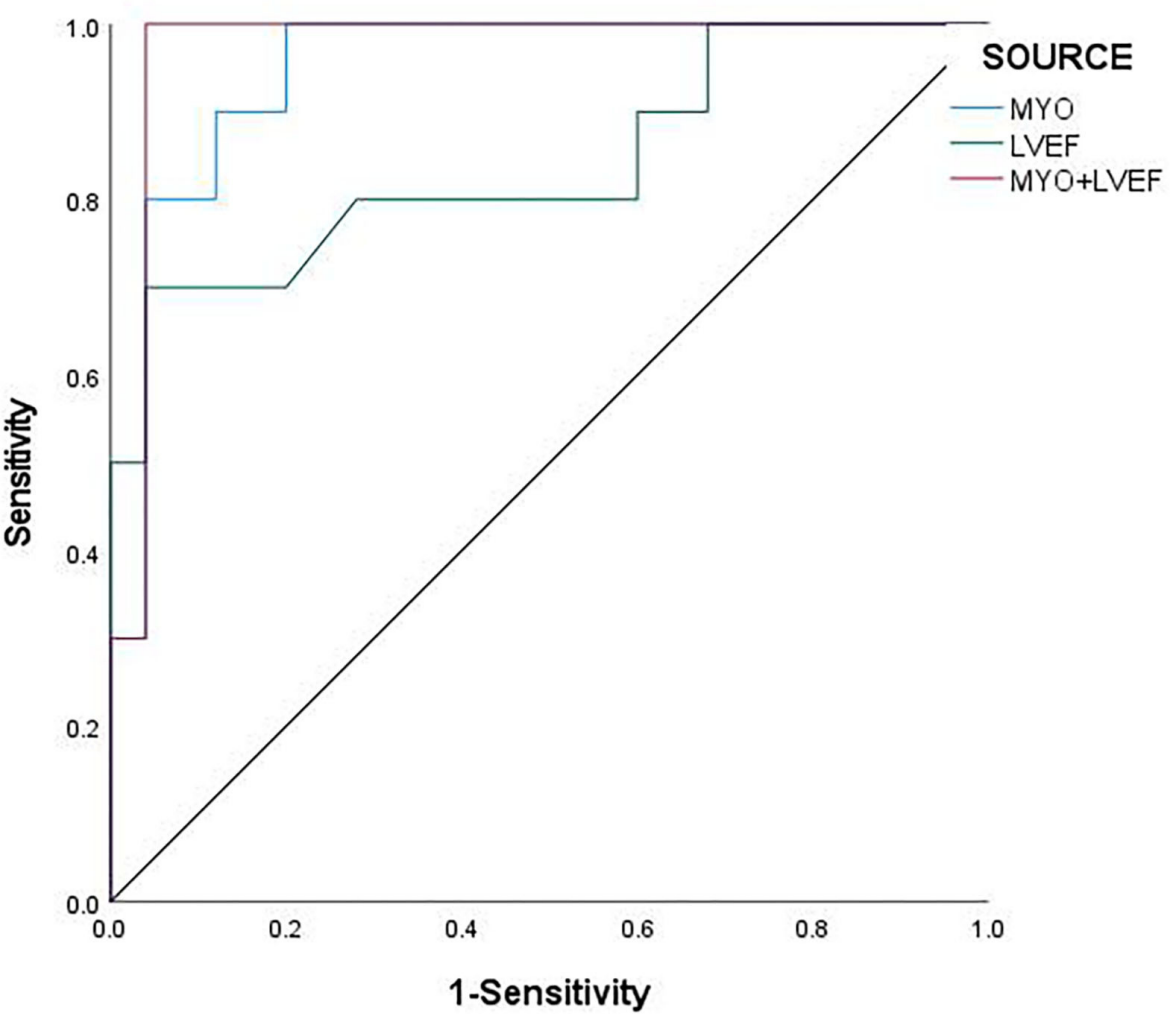
ROC curves of MYO, LVEF, and their combination predicting death in children with FM.

The ROC analysis revealed a cut-off value of MYO for FM of 210 μg/L, with a sensitivity of 91.7% and a specificity of 88% [AUC: 0.957; 95% CI: 0.897~1; *P* < 0.001], and Jorden Index was.797 (Figure 2).

Therefore, the patients were re-divided into two groups based on absolute myoglobin values. Low MYO (≤ 210, *n* = 23) indicated low risk of death, and high MYO (>210, *n* = 14) indicated high risk of death.

After adjusting for age and gender, the analysis showed that the high MYO group had more obvious damage to the liver, kidney, and cardiac function, and that the disease was more serious. In terms of outcomes, the low MYO group had a mortality of only 4.3%, while the high MYO group had a high mortality of 78.6% and a lower survival rate (Table 6).

**Table 6:** Comparison of clinical characteristics of patients with FM with different MYO levels.

**Characteristic**	**MYO ≤ 210** **(*n* = 23)**	**MYO>210** **(*n* = 14)**	***P***
LVEF [%, M (QI, Q3)]	44 (36, 59)	33 (20, 43)	0.031
AST [IU/L, M (QI, Q3)]	136.0 (74.5, 359.1)	423 (142.5, 1160.4)	0.110
CK-MB [IU/L, M (QI, Q3)]	69 (48, 105)	110.23 (63, 156.19)	0.058
CK [IU/L, M (QI, Q3)]	765 (604, 1,083)	1412 (1042, 2,233)	0.043
TNIU [μg/L, M (QI, Q3)]	10.08 (3.40, 28.06)	13.87 (6.81, 30.67)	0.459
ALT [IU/L, M (QI, Q3)]	42.3 (25, 89.8)	186.3 (37.1, 1008)	0.118
BUN [mmol/L, M (QI, Q3)]	6.4 (4.6, 10.4)	7.8 (4.9, 13.5)	0.392
CR [μmol/L, M (QI, Q3)]	50.8 (36, 64)	71.5 (53, 90.9)	0.114
Died [*n* (%)]	1 (4.3)	11 (78.6)	<0.001

The results suggested that patients in the high MYO group led to a greater rate of in-hospital death or transplantation and lower long-term survival compared to the low MYO group.

This study also analyzed the correlation between MYO and inflammatory markers such as PCT and CRP, which indicated PCT and CRP were positively correlated with MYO levels (Table 7).

**Table 7:** Correlation of MYO and inflammatory biomarkers in children with FM.

**Variables**	**Spearman** **Correlation**	**95%CI**	***P* value**
MYO-PCT	0.734	0.514~0.863	<0.001
MYO-CRP	0.373	0.023~0.641	0.033

## Discussion

An important feature of FM is the rapid clinical progression that can lead to cardiogenic shock and electrical instability, and requires inotropic or mechanical circulatory support. In China, there are no epidemiological statistics in the incidence of this disease. Previous foreign studies reported that the incidence of FM may account for approximately 10–38% of all acute myocarditis (11). A nationwide survey in Finland showed that the total incidence of myocarditis in children was 1.95/100,000 person-years from 2004 to 2014 (12). In addition, a similar nationwide survey in South Korea showed that the overall incidence of acute myocarditis was 1.4 per 100,000 children in 2007 and 2.1 per 100,000 children in 2016 (13). As a whole, we observed an increase in the incidence of acute myocarditis in children over the last decade. Recently, a Korean study showed that the survival rate of children with FM was 69.1%, which indicated that the mortality of children with FM was high. Overall, the high mortality of children with FM is a significant problem, given the growing prevalence of this challenging clinical syndrome.

The etiology of FM is not clear yet. Various etiological factors have been suggested to trigger FM. Relevant studies have shown that pathogen infection, immune disorders of autoimmune diseases, and drug toxicity can induce FM. By analyzing the cytokine profiles of patients with FM, in early stages of FM, various etiologies trigger inflammatory signals that lead to the transcription and translation of pro-inflammatory cytokines. We can find the up-and down-regulation of various cytokines, that is, the activation of a “cytokine storm” (14). Cytokine storms disturb immune homeostasis and can directly affect myocardial contraction and cardiac function (15). Cytokine storms play a key role in the pathogenesis of FM.

According to Xu et al. LAd [OR: 1.226; 95% CI: 1.016–1.478; *P* = 0.034] and eGFR level [OR: 0.928; 95% CI: 0.87–0.989; P = 0.022] were predictors of FM (6). Besides, a combination of high cTn levels and low BNP levels, which suggested poor outcomes of the patient with FM (16). Liu et al. found that the ACEF score, which was correlated with age, creatinine, and LVEF, [OR: 4.499; 95% CI: 0.960–1.061; *p* < 0.001] was a strong independent predictor of in-hospital mortality in children with FM, with an area under the ROC curve of.871 (7). A retrospective study in China analyzed the prognosis of 24 children with FM and found that LVEF reduction was a risk factor for poor prognosis in children with FM (17). However, it did not conduct a further analysis of the correlation between LVEF levels and FM.

In this retrospective study, MYO and LVEF were closely related to the prognosis of FM (*P* < 0.05). The ROC curve analysis showed that LVEF, MYO, and their combination index did well in predicting the clinical prognosis of children with FM and their AUCs were.957, 0.836, and 0.972, respectively. This current study found that the area under the combined ROC curve for MYO + LVEF was significantly higher than that for MYO or LVEF alone (*P* < 0.05), indicating that the MYO + LVEF combined diagnosis had a higher predictive value for FM as well as better sensitivity. In addition, we also found that the prognosis of children with different levels of MYO was significantly different. The mortality in the low MYO group was only 4.3%, while the mortality in the high MYO group was as high as 78.6%, which required more medical support and had lower survival rates. At the same time, we found that the increase of MYO was often accompanied by the increase of CRP and PCT, which may indicated that MYO was as important as some markers of myocardial injury. MYO was positively correlated with CRP and PCT.

Ejection fraction is the percentage of stroke volume in ventricular end-diastolic volume, and its decrease is often indicative of cardiac insufficiency. So, persistent myocardial damage in FM will lead to left ventricular dilation with systolic dysfunction (18, 19). As we all know, extensive or localized necrosis of cardiomyocytes or myocardial tissue, resulting results in a severe decrease in heart pumping function.

MYO is a cytoplasmic hemoglobin present in cardiomyocytes and skeletal muscle fibers (20), which facilitates oxygen transport and storage. The level of MYO is elevated by myocardial and skeletal muscle injury. In addition, MYO levels are elevated in diseases such as severe infection, severe trauma, and renal insufficiency (21, 22).

The myoglobin content in human myocardium is related to oxidative capacity (23). The release of MYO increases in the hypoxic environment. With aggravation of hypoxia, the level of MYO gradually increases. In the heart, under hypoxic conditions, MYO protects the heart against reactive oxygen species (ROS), and at the same time, deoxygenated MYO is able to reduce nitrite to NO, thereby reducing cardiac energy status and reducing cardiac damage after reoxygenation (24). In FM, infection with various pathogens induces a systemic inflammatory response, and decrease in cardiorespiratory function may lead to hypoxemia and decreased blood oxygen saturation. Simultaneously, in some patients, the blood pressure is too low due to severe hypoperfusion, which can also cause insufficient oxygen supply to the body. In this case, it is easy to cause difficulty breathing and thus hypoxia. Hypoxemia can lead to myocardial hypoxia, and chronic hypoxia can directly lead to myocardial injuries, resulting in myocardial dysfunction and hemodynamic disorders. Myocardial damage leads to increased MYO, while infection by various pathogens leads to the release of inflammatory factors, and cytokine storms exacerbate myocardial damage, apoptosis, and myocardial remodeling, creating a vicious cycle that leads to cardiac dysfunction.

Clinically rapid diagnosis of FM is essential to foster optimal treatments and outcomes for these children. Therefore, we believe that MYO and LVEF monitoring should be carried out for children with FM as soon as possible. A sharp increase in MYO level and a marked decrease in LVEF, especially when MYO >210 μg/L and LVEF <31.5%, are an important warning. It is helpful for clinicians to judge the follow-up treatment, establish circulatory support, and maintain end-organ function as soon as possible (25), which has great support for reducing the high mortality of FM.

This study was retrospective with a small sample size and lacked support from cardiac MRI. There were certain limitations, which need to be further verified with large data samples.

## Conclusion

Monitoring of MYO and LVEF levels on admission may serve as a potential biomarker for assessing the severity of FM and further predicting death.

## Data availability statement

The raw data supporting the conclusions of this article will be made available by the authors, without undue reservation.

## Ethics statement

The studies involving human participants were reviewed and approved by the hospital Ethics Committee of Anhui Provincial Hospital and Children's Hospital of Nanjing Medical University. Written informed consent to participate in this study was provided by the participants' legal guardian/next of kin.

## Author contributions

TX has obtained the approval of XZ, YX, CY, FL, DW, YS, XM, and MC to submit this article. All authors have contributed to the manuscript. All authors contributed to the article and approved the submitted version.

## Funding

This study was supported by the Natural Science Foundation of Anhui Province (Grant No: 21608085MH196) and the Ministry of Science and Technology of the People's Republic of China (Grant No: 2018YFC1003700).

## Conflict of interest

The authors declare that the research was conducted in the absence of any commercial or financial relationships that could be construed as a potential conflict of interest.

## Publisher's note

All claims expressed in this article are solely those of the authors and do not necessarily represent those of their affiliated organizations, or those of the publisher, the editors and the reviewers. Any product that may be evaluated in this article, or claim that may be made by its manufacturer, is not guaranteed or endorsed by the publisher.
